# Supplementary material for: Construction and iterative redesign of synXVI a 903 kb synthetic Saccharomyces cerevisiae chromosome
Source: Nat Commun. 2025 Jan 20;16:841. doi: 10.1038/s41467-024-55318-3 (PMC11747415; doi:10.1038/s41467-024-55318-3)
Supplement: Supplementary file 1 — Supplementary Information [file 41467_2024_55318_MOESM1_ESM.pdf]

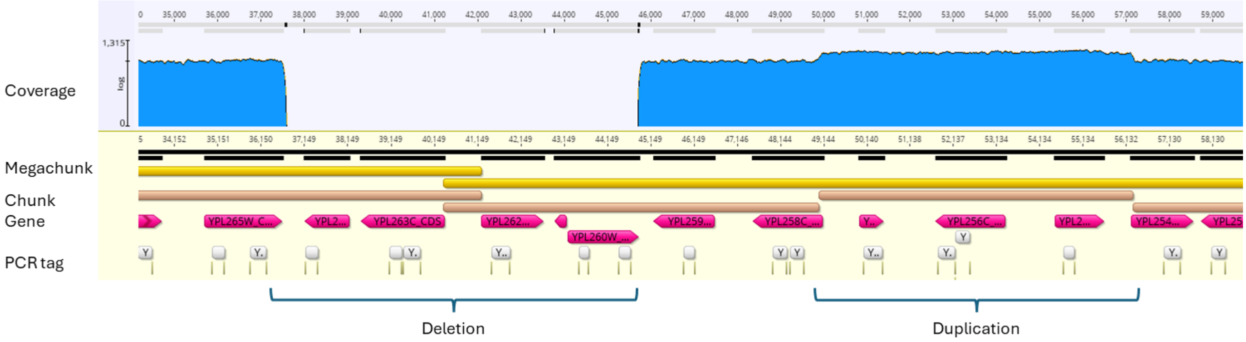

Supplementary Figure 1: An example of sequencing read depth visualised in Geneious 9.1.8 (Biomatters). Paired reads were joined and mapped to the synXVI genome sequence using Geneious mapper. Mean coverage was 221 reads. In the locus labelled deletion mean read coverage was 0. In the locus labelled duplication mean read coverage was 468.5. Similar approaches were used to identify loss of mitochondrial genome, chunk and megachunk duplication, chromosomal insertion of pUC vectors in various strains included and omitted from the study.

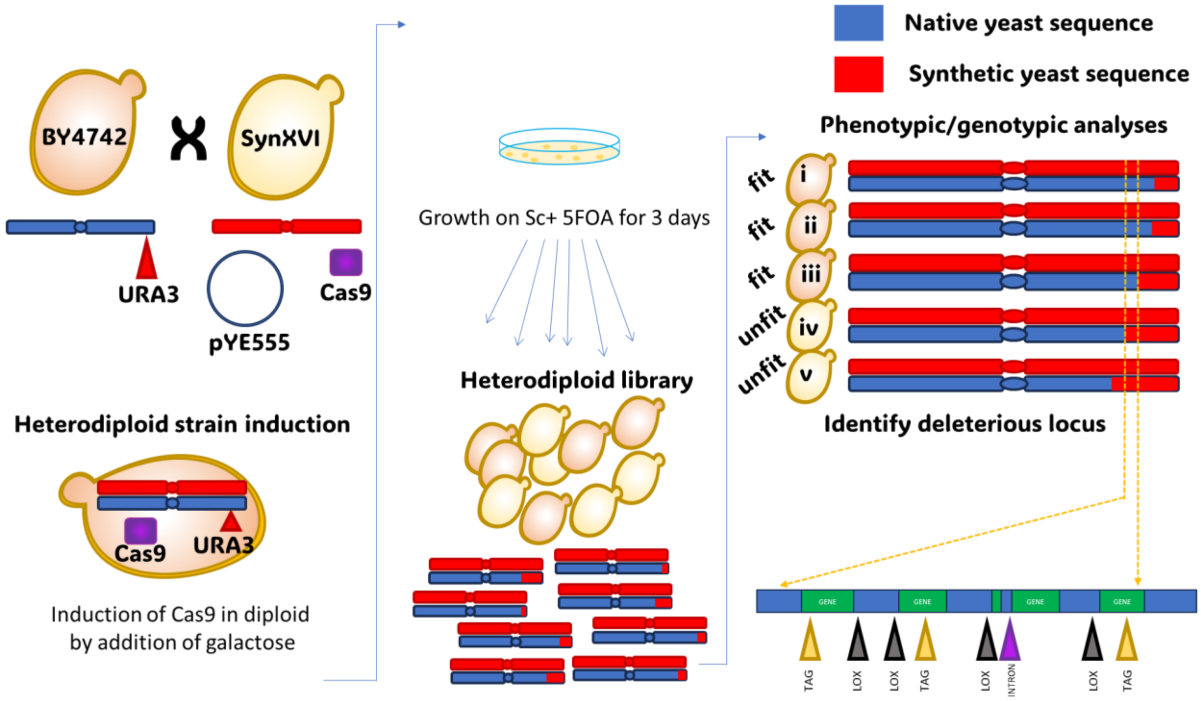

Supplementary Figure 2: Explanation of CRISPR-D-BUGS deployment

URA3 markers were inserted in telomeric loci of BY4742 (strains 25 and 26). BY4742 was mated with defective strains bearing the defective synthetic chromosome XVI (strains 27 and 28). Diploid cells bearing one wildtype chromosome with a *URA3* marker adjacent to the telomere, and one synthetic chromosome, as well as pYE555 encoding 0020a gRNA recognising the site on the wild-type chromosome corresponding to a PCR tag across the chromosome (full list of PAM sites in supplementary table 1). Cas9 production was induced by

overnight growth in galactose, and chromosome arm crossovers were selected for using 5FOA. Resulting strains were phenotypically analysed using spot assays. Segregation of phenotypes into health and unhealthy on YP-Glycerol or at 37˚C, or under both conditions, allowed for identification of defective loci.

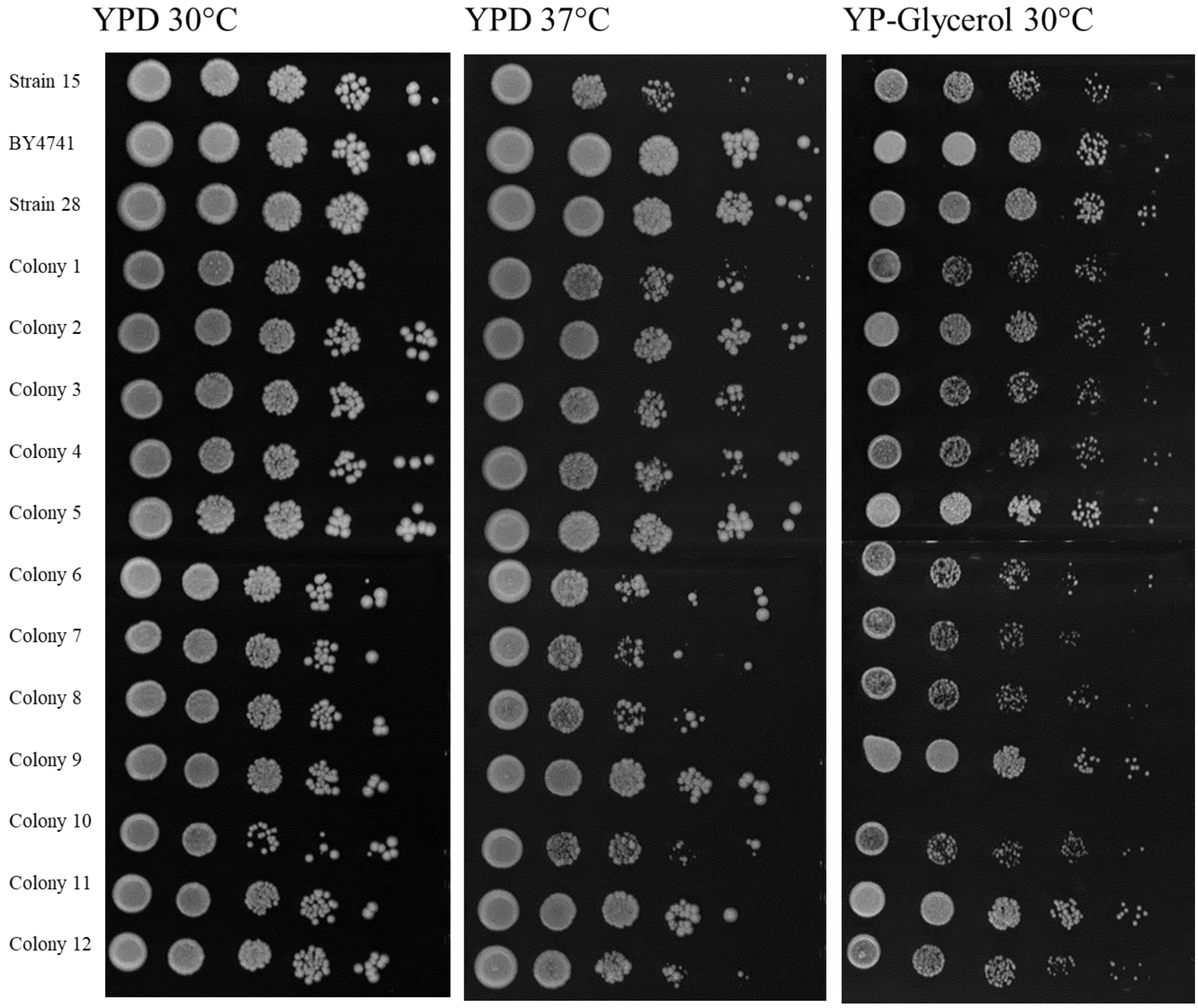

Supplementary Figure 3: Spot assays showing CTR1 phenotype

The CRISPR D-BUGS protocol was applied to a diploid strain bearing a *URA3* marker adjacent to the R telomere (strain 28), with a pYE555 plasmid encoding a gRNA targeted to X3. Twelve individual colonies were grown overnight and then plated on YPD at 37˚C, and on YP Glycerol for comparison to BY4741, a diploid bearing a wildtype chromosome and the *synXVI* chromosome, relative to BY4741 and the hybrid diploid, *synXVI* demonstrates poor growth. Colonies 11, 9 and 5 demonstrate improved fitness on YP Glycerol at 30˚C.

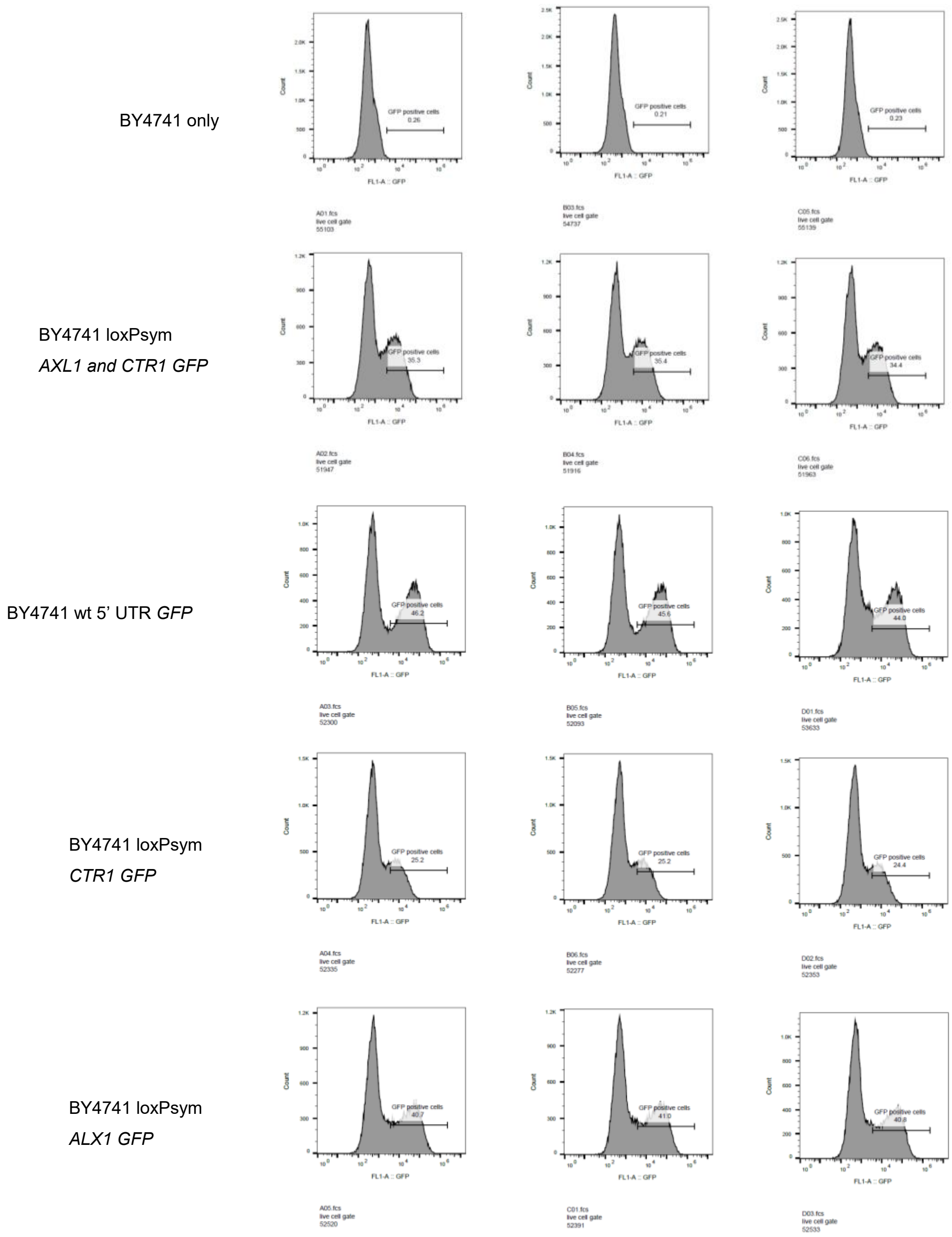

Supplementary Figure 4: GFP reporter assay of CTR1 and GIP3 native and synthetic promoters.

Raw data of GFP report assay showing proportion of GFP + BY4741 cells harbouring plasmids encoded with synthetic, wildtype, and mixed promoters and 5’ UTR of CTR1 and GIP3.

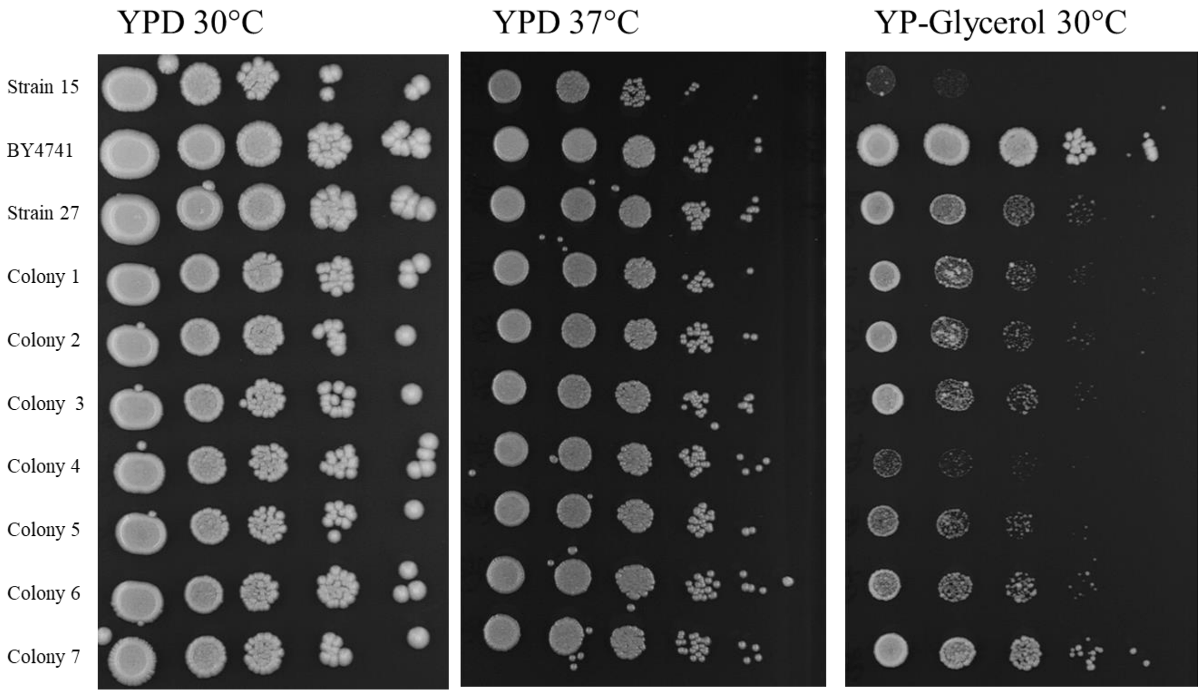

Supplementary Figure 5: Spot assays showing GIP1 phenotype

The CRISPR D-BUGS protocol was applied to a hybrid diploid strain bearing a *URA3* marker adjacent to the L telomere (strain 27), with a pYE555 plasmid encoding a gRNA targeted to J3. Seven single colonies were grown overnight then plated on YPD at 37˚C, and on YP Glycerol for comparison to BY4741, strain 27, a diploid bearing a wildtype chromosome and the *synXVI* chromosome. Relative to BY4741 and the hybrid diploid, *synXVI* demonstrates poor growth, indicating that all the fitness defects are recessive. Colony 7 demonstrated improved fitness on YP Glycerol at 30˚C.

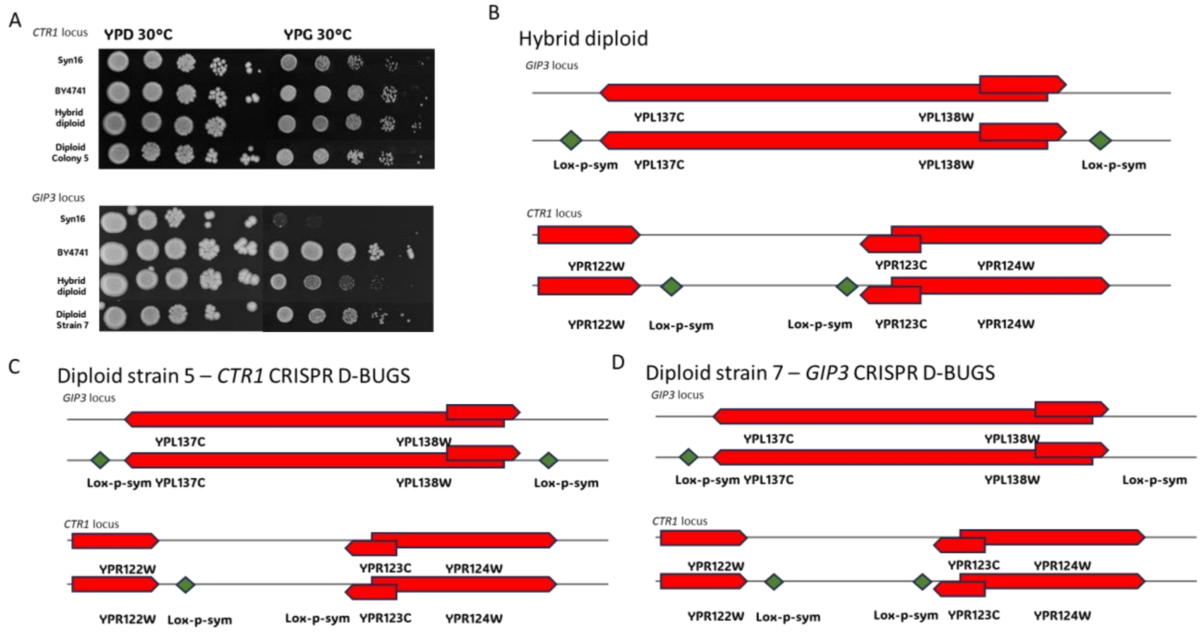

Supplementary Figure 6: Hybrid diploids from spot assays (A) and schema demonstrating fitness genome sequencing data for colony 5 for CRISPR D-BUGS Z, as well as colony 7

for CRISPR D-BUGS J. In the hybrid diploid (B), one copy of the BY4742 chromosome exists, and one copy of the synthetic strain 15 chromosome exists. The crossovers initiated by CRISPR D-BUGS resulted in removal of the loxPsym site causing defective growth in the locus adjacent to the effected genes (panel c for CTR1, and panel d for GIP3), improving growth of the resulting hybrid diploids. For a full example of the phenotypes analysed Supplementary Figure 3 and 5 show all phenotypes resulting from CRISPR-DBUGS Z and J.

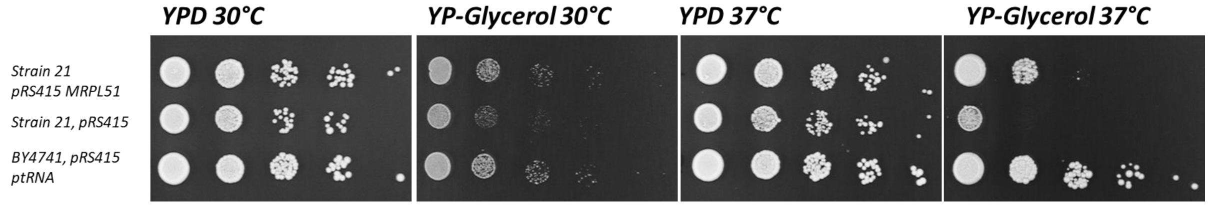

Supplementary Figure 7: Partial complementation of a loxPsym site defect associated with mitochondrial dysfunction

Given the demonstrated pattern of loxPsym sites existing in 5' UTR sequences of genes with overlapping putative ORFs, *MRPL51* was identified as a likely candidate for a growth defect. Strains grown with wild-type *MRPL51* encoded on a pRS415 vector had growth similar to wild-type colonies when grown at 30˚C on YP medium containing glycerol as a nonfermentable carbon source. Photos on YPD at 30˚C and 37˚C, and YP-Glycerol at 30˚C were taken at 3 days, and photos on YP-Glycerol at 37˚C were taken at 6 days.

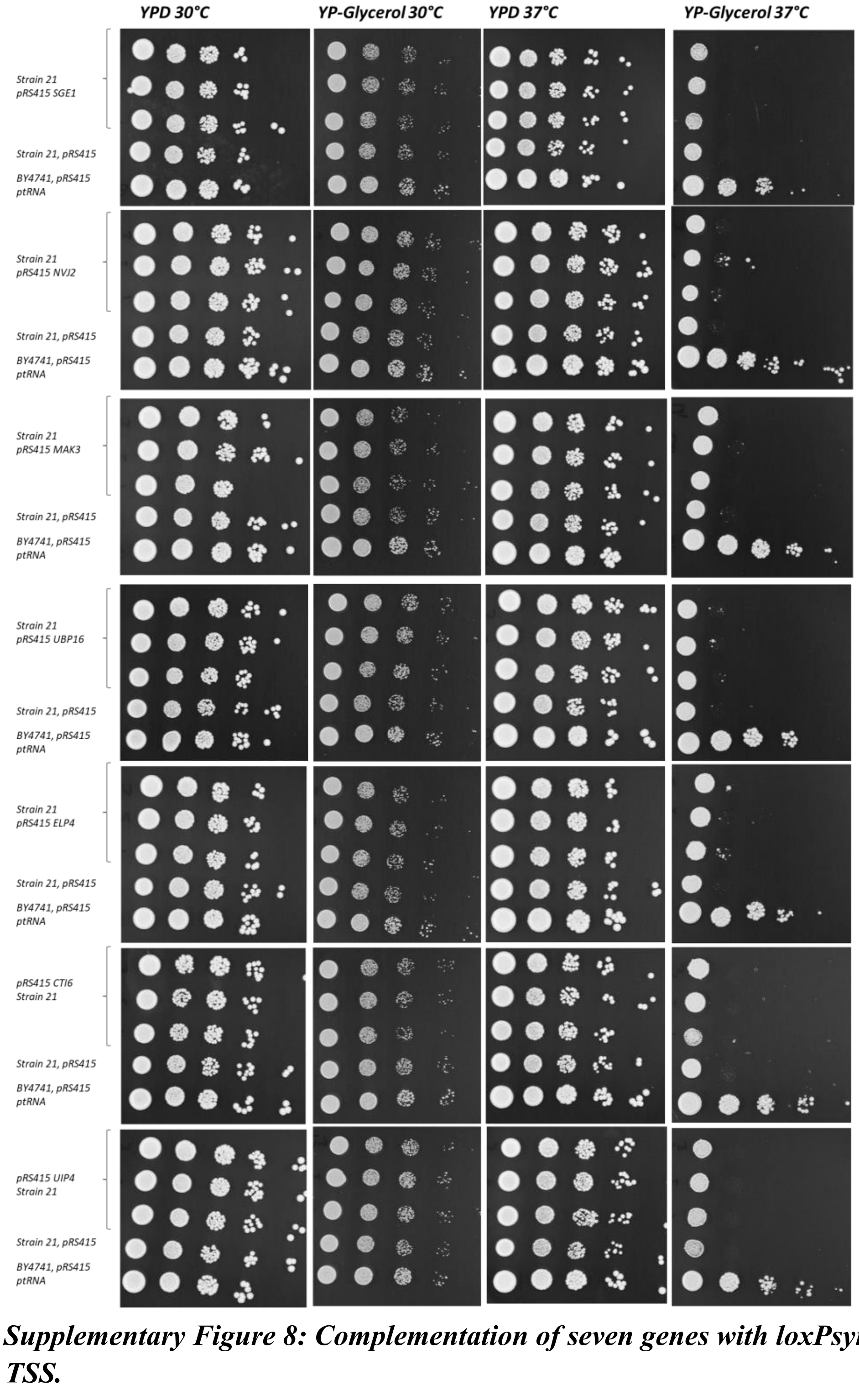

Supplementary Figure 8: Complementation of seven genes with loxPsym sites proximal to TSS.

Spot assays were conducted to compare strain 21 with wildtype genes *SGE1, NVJ2, MAK3, UBP16, CTI6, ELP4,* and UIP4 expressed under native regulation on pRS415, with Strain 21 with a pRS415 empty vector, and BY4741 with pRS415 and pRS413. Photos on YPD at 30˚C and 37˚C, and YP-Glycerol at 30˚C were taken at 3 days, and photos on YP-Glycerol at 37˚C was taken at 6 days. The results suggest that none of these genes is a mjor contributor to fitness defects observed in *synXVI* strain 21.

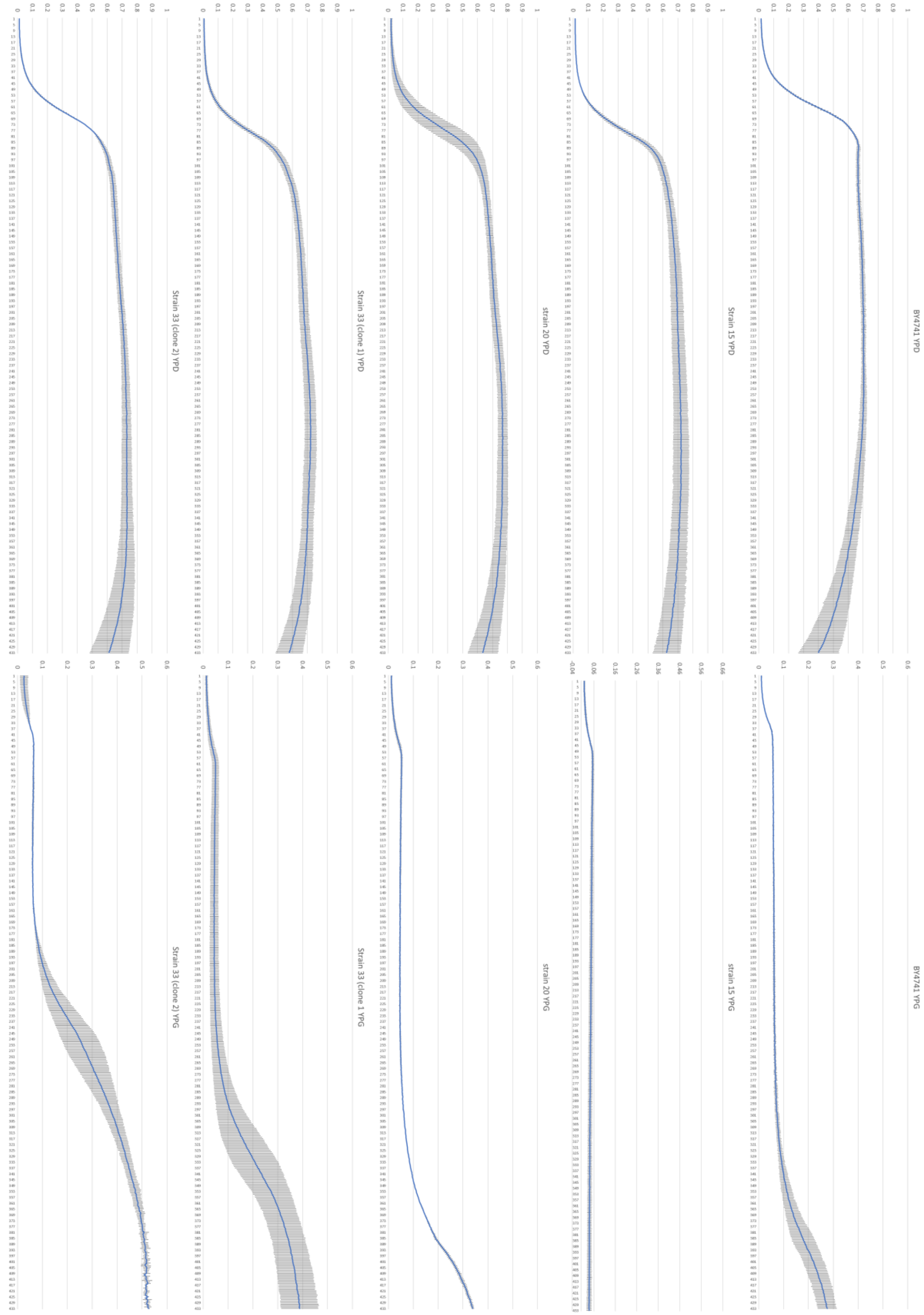

Supplementary Figure 9: Growth of curves of synXVI bearing strains compared to wildtype.

BY4741, Strain 15, Strain 20 and two clones of Strain 33 grown at 30°C in YPD or YPGlycerol, OD600 measurements (y-axis) were taken in 15-minute intervals over 48 hours (x-axis), standard deviation from the mean of three biological replicates is shown as error

bars. Source data are provided as a Source Data file. For comparison the maximum rate of change at OD_{600} per hour for BY4741, strain 15, strain 20, strain 33 clone 1, and clone 2, in YPD are 0.126, 0.104, 0.1, 0.1 and 0.112, and 0.03 0.012, 0.036, 0.032 and 0.058 in YPGlycerol respectively.

AMINO ACID	ANTICODON	NUCLEAR GENOME REDUNDANCY	NAME	SYSTEMATIC NAME
**ALANINE**	AGC	11	tRNA-Ala	tA(AGC)P
**GLUTAMATE**	UUC	14	tRNA-Glu	tE(UUC)P
**PHENYLALANINE**	GAA	10	tRNA-Phe	tF(GAA)P2
**GLYCINE**	GCC	16	tRNA-Gly	tG(GCC)P1
**LYSINE**	UUU	7	tRNA-Lys	tK(UUU)P
**TRYPTOPHAN**	CCA	6	tRNA-Trp	tW(CCA)P
**CYSTEINE**	GCA	4	tRNA-Cys	tC(GCA)P1
**CYSTEINE**	GCA	4	tRNA-Cys	tC(GCA)P2
**PHENYLALANINE**	GAA	10	tRNA-Phe	tF(GAA)P1
**GLYCINE**	GCC	16	tRNA-Gly	tG(GCC)P2
**ISOLEUCINE**	AAU	13	tRNA-Ile	tI(AAU)P1
**ISOLEUCINE**	AAU	13	tRNA-Ile	tI(AAU)P2
**LYSINE**	CUU	dfd	tRNA-Lys	tK(CUU)P
**ASPARAGINE**	GUU	10	tRNA-Asn	tN(GUU)P
**THREONINE**	UGU	4	tRNA-Thr	tT(UGU)P
**METHIONINE (INITIATION)**	CAU	4*	tRNA-Met	tM(CAU)P
**SERINE**	UGA	3	tRNA-Ser	tS(UGA)P

Supplementary Table 2: A complete list of 17 tRNA molecules removed from chromosome 16 by design

Chunk	Dubious ORF	Verified ORF	Verified ORF name	Distance to overlap loxPsym	Approximate TSS distance	Annotated function of verified ORF
F3	*YPL185W*	*YPL186C*	*UIP4*	225bp	210bp	Protein required for nuclear envelope integrity
F4	*YPL182C*	*YPL181W*	*CTI6*	118bp	122bp	Component of the Rpd3L histone deacetylase complex
I4	*YPL136W*	*YPL137C*	*GIP3*	10bp	509bp	Cytoplasmic protein that regulates protein phosphatase 1 Glc7p
K4	*YPL102C*	*YPL101W*	*ELP4*	209bp	51bp	Subunit of hexameric RecA-like ATPase Elp456 Elongator subcomplex

M4	*YPL073C*	*YPL072W*	*UBP16*	16bp	190bp	Putative deubiquitinating enzyme anchored to the outer mitochondrial membrane
U1	*YPR050C*	*YPR051W*	*MAK3*	11bp	54bp	Catalytic subunit of the NatC type N-terminal acetyltransferase (NAT)
V3	*YPR092W*	*YPR091C*	*NVJ2*	219bp	52bp	Lipid-binding ER protein
W1	*YPR099C*	*YPR100W*	*MRPL51*	37bp	38bp	Mitochondrial ribosomal protein of the large subunit
X4	*UPR123C*	*YPR124W*	*CTR1*	70bp	219bp	High-affinity copper transporter of plasma membrane;
BB3	*YPR197C*	*YPR198W*	*SGE1*	139bp	55bp	Plasma membrane multidrug transporter

Supplementary Table 3: List of overlapping dubious ORFs and the genes they interrupt, with functional annotations from the Saccharomyces genome *database.*

Transcription start sites (TSS) interrupted by insertion of a loxPsym site to a dubious ORF are highlighted in red. Verified ORF names which were detected in proteomic analysis are in red.
