# Supplementary material for: Construction and iterative redesign of synXVI a 903 kb synthetic Saccharomyces cerevisiae chromosome
Source: Nat Commun. 2025 Jan 20;16:841. doi: 10.1038/s41467-024-55318-3 (PMC11747415; doi:10.1038/s41467-024-55318-3)
Supplement: Supplementary file 3 — Description of Additional Supplementary Files [file 41467_2024_55318_MOESM3_ESM.pdf]

Description of Additional Supplementary Files

Supplementary Data 1: Supplementary tables including supplementary Table 1, a list of PAM sites used in CRISPR DBUGS, Supplementary Table 5, a list of genes interrupted by the SwAP-IN protocol, and assignment of LEU2 or URA3 markers interrupting coding sequences with annotated deleterious phenotypes of the interrupted genes from the Saccharomyces Genome Database, supplementary table 6, a list of strains used in this study, Supplementary table 7 a list of vectors used in the construction of synXVI, supplementary table 8 which is a list of genotypic details of major synXVI strains detailed in this study and supplementary Table 9 a list of oligonucleotides used for tag analysis in this study
